# Comparison of Oral Health Impact Profile (OHIP-14) Values in Cancer Survivor Patients Treated Orthodontically with Either Rapid or Standard Duration Protocols of Treatment—A Prospective Case–Control Study

**DOI:** 10.3390/ijerph17239068

**Published:** 2020-12-04

**Authors:** Maria Mitus-Kenig, Marcin Derwich, Ewa Czochrowska, Elzbieta Pawlowska

**Affiliations:** 1 Department of Experimental Dentistry and Prophylaxis, Medical College, Jagiellonian University in Krakow, 31-008 Krakow, Poland; maria.mitus@interia.pl; 2 ORTODENT, Specialist Orthodontic Private Practice in Grudziadz, 86-300 Grudziadz, Poland; 3 Department of Orthodontics, Medical University of Warsaw, 02-091 Warsaw, Poland; info@czochrowska.com; 4 Department of Orthodontics, Medical University of Lodz, 90-419 Lodz, Poland; elzbieta.pawlowska@umed.lodz.pl

**Keywords:** oral-health-related quality of life, oral health impact profile, OHIP-14, cancer survivors

## Abstract

Background: The aim of the study was to compare the oral-health-related quality of life (OHRQoL) between cancer survivors: with rapid orthodontic treatment (TX) (up to 12 months) and standard TX (orthodontic treatment time longer than 16 months). Methods: There were 76 cancer survivors (48 women and 28 men) allocated into groups with rapid (36 people) or standard (40 people) duration of TX. OHRQoL was assessed on the basis of Oral Health Impact Profile (OHIP-14) values, measured before TX, 2 weeks and 3 months after the onset of TX, and after the end of TX. A repeated ANOVA test was used to check the statistical significance between the scores. Results: There were no significant differences regarding the OHIP-14 values between the examined groups at all stages of the examination. Both groups presented significant (*p* < 0.001) improvement of the values of OHRQoL at the end of TX comparing to the values achieved before the onset of TX. Conclusions: Duration of orthodontic treatment by itself had no impact on oral-health-related quality of life.

## 1. Introduction

“Cancer survivor” is a general term describing an adolescent or adult patient who was diagnosed with cancer and successfully underwent oncological treatment. Although the exact number of children diagnosed with cancer each year is unknown, the statistics present the increase in the incidence rates of cancers among children [1,2]. Children are most often diagnosed with leukemias, lymphomas, and tumors of central nervous system [1,2]. The cancer treatment modalities encompass chemotherapy, radiotherapy, surgery, and a combination of the above-mentioned methods [3]. Because of better understanding of the cancer biology and thanks to the advances in oncological treatment, the 5-year survival rates have been improved. Nowadays, the 5-year survival rates have achieved the value of 80% [4,5]. The European country-weighted 5-year survival assessed for patients diagnosed with cancer from 2000 to 2007 presented as follows: acute lymphoid leukemia—86.3%, acute myeloid leukemia—62.7%, Hodgkin’s lymphoma—95.4%, non-Hodgkin lymphoma (except Burkitt’s lymphoma)—84.0%, Burkitt’s lymphoma—90.2%, Central Nervous System (CNS) and miscellaneous intercranial and intraspinal neoplasms—57.5%, neuroblastoma and ganglioneuroblastoma—70.6%, retinoblastoma—96.4%, nephroblastoma and other nonepithelial renal tumors—89.4%, osteosarcomas—69.3%, Ewing’s sarcoma and related sarcomas of bone—67.9%, rhabdomyosarcomas—67.7% [4]. Therefore, the number of cancer survivors is continuously increasing. According to the literature, one out of 900 young adults received successful oncological treatment in their childhood [6]. As a consequence, there is also an increase in the number of cancer survivors seeking orthodontic treatment.

It was assessed that 72% of orthodontists who had treated cancer survivors reported the presence of some dental complications that occurred as consequences of oncological treatment. These dental complications included the following: malaligned teeth (22%), root stunting (17%), growth and development changes (16%), missing teeth (13%), delay in loss of deciduous teeth (12%), microdontia (11%), and enamel hypoplasia (9%) [7,8]. Moreover, irradiation in the area of the head and neck may lead to oral mucositis, dysfunctional taste, and malnutrition as well as radiation caries [9]. Because of this fact, that most of the cancer survivors presented some dental complications, and the majority of orthodontists (75%) prescribed some modifications to orthodontic treatment [7]. The American Academy of Pediatric Dentistry (AAPD) recommended five different strategies for the orthodontic treatment of cancer survivors, including the following: usage of the appliances that reduce the risk of root resorption, usage of lighter forces, choosing the simplest method for the treatment needs, not to treat the lower arch, and finally to terminate the treatment earlier than normal [10].

Estimated orthodontic treatment time is one of the most important pieces of information for the patients. Patients, who finish their treatment on time, seem to be more satisfied [11]. There are many factors that affect estimated treatment time [11]. One of these, that should be considered, is previous oncological treatment. Cancer survivors may need rapid orthodontic treatment because of the planned oncological follow-up or maintenance therapy. Moreover, their orthodontic treatment sometimes has to be suspended for a while.

One of the major goals of orthodontic treatment in cancer survivors is the improvement of patients’ quality of life. The quality of life is a complex term encompassing individuals’ perception of different aspects of life. The World Health Organization defined quality of life as an “individual’s perception of their position in life in the context of the culture and value systems in which they live and in relation to their goals, expectations, standards and concerns. It is a broad ranging concept affected in a complex way by the person’s physical health, psychological state, personal beliefs, social relationships and their relationship to salient features of their environment” [12,13]. Oral-health-related quality of life (OHRQoL) measures how oral diseases or conditions related to the oral cavity influence the patient’s life [14]. It has been proven that malocclusions leading to severely compromised esthetics have negative impact on OHRQoL [15], which improves after the end of orthodontic treatment [16]. However, the initial phases of orthodontic treatment have been found to have a negative impact on OHRQoL [17]. Unfortunately, little is known about the influence of orthodontic treatment on OHRQoL in the group of cancer survivors [18].

Therefore, the aim of the study was to compare prospectively the oral-health-related quality of life between two groups of cancer survivors: those who had undergone rapid orthodontic treatment (up to 12 months) and standard orthodontic treatment (orthodontic treatment time longer than 16 months) from 2012 till 2016 in Krakow (Poland).

## 2. Materials and Methods

The Medical Board Ethical Committee (50/KBL/OIL/2010) approved the study. This research was conducted with the ethical principles of the World Medical Association Declaration of Helsinki. Informed consent was received and signed by all the patients. Parents received a letter describing the study protocol and requesting consent for their children to participate in the study.

### 2.1. Study Population

The patients from both groups were selected and treated in the same specialist orthodontic private practice in Krakow (Poland). There were 81 participants (50 women and 31 men; median age: 19.3; age range: 13–28 years) enrolled into the study. All the patients had successfully undergone oncological treatment in their childhood due to cancer and came to the specialist orthodontic practice looking for orthodontic treatment. The exclusion criteria were as follows: previous orthodontic treatment, severe dentofacial anomalies, poor periodontal health, and patients who did not agree to take part into the study.

Before the onset of orthodontic treatment, each patient was referred for a specialist oncological consultation. Having received the relevant information from the oncologists, patients were allocated into two groups with rapid and standard duration of orthodontic treatment. Patients with the risk of possible hospitalization, magnetic resonance imaging examination, or any other types of oncological examinations that could have influenced orthodontic treatment within the following one year, were classified to the rapid orthodontic group. The orthodontic treatment in the rapid group was expected to be completed within the next 12 months. The remaining patients were classified as the control group or the group with standard duration of orthodontic treatment. The control group was not limited by the duration of the orthodontic treatment.

**Figure 1:**
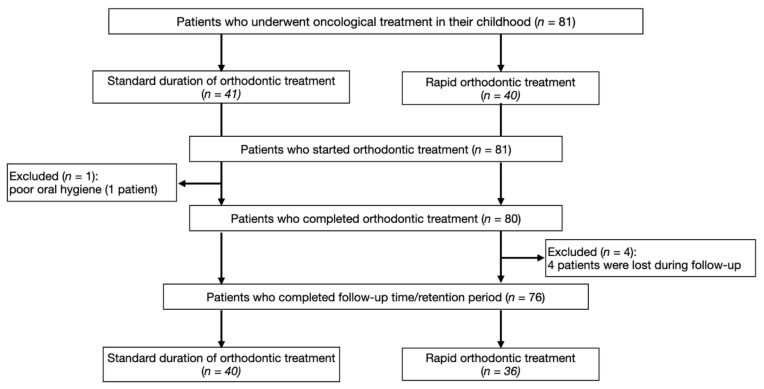
The flow chart of participation diagram.

The flow chart of participation is presented in Figure 1. Table 1 presents the general characteristics of the cancer survivors and the control groups.

Table 2 presents the general characteristics of both rapid and standard groups with reference to the diagnosed type of cancer, including mean age at diagnosis, follow-up time, and treatment modality.

### 2.2. Study Protocol

This was a prospective case–control study. The patients from both groups (with rapid and standard duration of orthodontic treatment) were selected and treated in the same specialist orthodontic private practice in Krakow (Southern Poland). The process of standard orthodontic diagnosis was based on extraoral and intraoral orthodontic examination, analysis of extraoral and intraoral photographs, plaster casts analysis, lateral cephalogram analysis, and dental panoramic tomogram analysis. All the records were collected prospectively. The orthodontic diagnosis and qualification to orthodontic treatment were performed by two independent certified specialists of orthodontics [18].

The orthodontic treatments of all participants were performed with vestibular fixed orthodontic appliances both in the maxilla, as well as in the mandible. We used traditional, ceramic, twin brackets with a 0.022 inch bracket slot and with MBT (McLaughlin/Bennett/Trevisi) prescription. Sliding mechanics was used in both groups. There was no need for the usage of skeletal anchorage devices. Intermaxillary elastics and the Goshgarian transpalatal bar were enough to gain satisfactory anchorage.

The goals of the orthodontic treatment were to achieve ideal occlusion as defined by Andrews [19], well-balanced facial profile, and to avoid complications that could have occurred in post-oncological-treatment patients. The primary endpoint of the study was to assess the influence of orthodontic treatment on patients’ health-related quality of life regarding the duration of the treatment.

The oral-health-related quality of life was assessed on the basis of the 14-item Oral Health Impact Profile (OHIP-14) Questionnaire [16,20]. The OHIP-14 Questionnaire was translated into the Polish language, following the principles recommended by the World Health Organization (forward translation, expert panel back-translation, pre-testing and cognitive interviewing, and final version) [21]. The OHIP-14 Questionnaire consists of 7 domains, namely, functional limitation, physical pain, psychological discomfort, physical disability, psychological disability, social disability, and handicap. There are two questions in each domain. The five-point Likert scale was used for rating patients’ responses. The answers were coded with the numbers: 0 (=never), 1 (=hardly ever), 2 (=occasionally), 3 (=fairly often), and 4 (=very often/every day) [22]. To score the answers marked by the patients, we used two different methods: the OHIP-14 additive method and the OHIP-14 simple count method. In the OHIP-14 additive method, the total score was achieved by summing the ordinal values for the 14 above-mentioned questions (the minimum score was 0 and the maximum score was 56). In the OHIP-14 simple count method, the final result was calculated by summing up the number of domains reported as occasionally or more frequently (therefore the total score ranged from 0 to 7). The OHIP-14 Questionnaires were filled in by the patients several times: before orthodontic treatment, 2 weeks after the onset of orthodontic treatment, 3 months after the onset of orthodontic treatment, and finally in the retention period. Table 3 presents the OHIP-14 Questionnaire. Pre-treatment values of the OHIP-14 were used as control ones.

### 2.3. Statistical Analysis

The data were analyzed using Statistica 13.0 software (Dell Inc., Aliso Viejo, CA, USA). No data were missing. Categorical variables were described as percentages of the total population, while continuous variables were reported as median and range. The Pearson’s chi-square or Fisher’s exact test was used to compare categorical variables. The Shapiro–Wilk and the Kolmogorov–Smirnov tests, with the Lilliefors correction, were used to confirm the normality of the distribution of the continuous variables. Finally, the unpaired Student t test or the nonparametric Mann–Whitney U test was used for comparisons. The ANOVA with repeated responses was used to assess differences in OHIP-14 across groups and time of treatment. Statistical significance was set at *p* ≤ 0.05.

## 3. Results

We calculated the required sample size considering the results of a pilot group, 90% of power and type I error of 0.05. To detect 20% difference between the groups in the OHIP-14 index after the treatment, the study sample in each group should include at least 34 patients.

Finally, there were 76 participants (48 women and 28 men; median age: 19.4, age range: 13–28 years) included in the study, because of the fact that one patient was excluded due to poor oral hygiene and four patients were lost during follow-up. There were 24 patients with skeletal class I malocclusion, 42 patients with skeletal class II malocclusion, and 10 patients with skeletal class III malocclusion (Table 1).

The vast majority of the patients in both groups had been diagnosed with leukemia (66.67% in rapid group vs. 55.0% in standard group). The frequency of other types of diagnosed cancers presented as follows: neuroblastoma (11.11% in rapid group vs. 7.50% in standard group), soft tissue sarcoma (2.78% in rapid group vs. 7.50% in standard group), non-Hodgkin’s lymphoma (19.44% in rapid group vs. 15.0% in standard group). Patients diagnosed with Wilms’ tumor were treated orthodontically only in the standard group (15.0%) (Table 2).

All of the patients had been treated in their childhood with chemotherapy. There were four patients who had received additional radiotherapy (two patients in each group), but none of them developed osteoradionecrosis. However, one of the patients needed hyperbaric oxygen support after the tooth extraction (Table 2).

Having analyzed dental panoramic tomograms, root resorption was diagnosed in four patients from the standard group. None of the patients from the rapid group presented any signs of root resorption. In all, 13 patients in the whole study suffered from oral mucositis (seven patients in the standard group vs. six patients in the rapid group). Moreover, 14 patients had their appliances temporarily removed to perform magnetic resonance imaging examinations (five patients in the rapid group vs. nine patients in the standard group).

The mean orthodontic treatment time was significantly shorter (*p* < 0.01) in the rapid group (11.3 months) comparing to that in the standard group (19.3 months). The mean follow-up time was similar in both groups and lasted 24.6 months.

The final results of orthodontic treatment, assessed on the basis of plaster casts, cephalometric images, and photographic documentation, presented no differences between the examined groups. However, there were four patients in the rapid group and two patients in the standard group who did not achieve the ideal occlusion at the end of orthodontic treatment.

### 3.1. OHIP-14 Mean Total Score

The OHIP-14 Mean Total Score values changed significantly throughout the orthodontic treatment in both groups. Two weeks and 3 months after the onset of orthodontic treatment, the OHIP-14 Mean Total Score values were significantly higher (*p* < 0.001) comparing to the values obtained before the orthodontic treatment, both in the rapid and the standard groups. However, after the end of orthodontic treatment, the OHIP-14 Mean Total Score values were significantly lower (*p* < 0.001) in both groups comparing to the values obtained before the onset of orthodontic treatment. Although the values of the OHIP-14 Mean Total Score differed between the examined groups, the differences were statistically insignificant.

Table 4 presents the OHIP-14 Mean Total Score in the cancer survivors treated orthodontically according to the rapid and standard protocols, before, during, and after the orthodontic treatment.

Having analyzed individual domains of the OHIP-14 score (functional limitation, physical pain, psychological discomfort, physical disability, psychological disability, social disability, and handicap), there were no statistically significant differences (*p* > 0.05) between the examined groups at all stages of the examination: before, during, and after the end of orthodontic treatment. Table 5 presents the mean OHIP-14 scores for individual domains observed before, during, and after orthodontic treatment for the two groups of cancer survivors: with the rapid and the standard duration orthodontic treatment.

### 3.2. OHIP-14 Simple Count

There were no statistically significant differences between the examined groups regarding the number of patients with impaired domains. Table 6 presents the number of patients with impaired domains in cancer survivors with rapid and standard duration of orthodontic treatment.

The number of patients with oral health impacts reported as occasionally or more frequently on the basis of OHIP-14 Simple Count did not differ significantly between the two examined groups. Table 7 presents the number and percentages of patients with oral health impacts reported as occasionally or more frequently, assessed on the basis of the OHIP-14 Simple Count.

## 4. Discussion

Assessment of the oral-health-related quality of life (OHRQoL) with reference to orthodontic treatment has been discussed by many authors [15,16,17,18]. Although there are many different methods to measure OHRQoL, one of the most commonly used is the Oral Health Impact Profile (OHIP). The 14-question, shorter version of the OHIP survey presents good reliability and validity [16]. Therefore, we decided to use the OHIP-14 survey to assess OHRQoL among all of the patients included in the study. Our study was the first one that prospectively compared OHRQoL between two groups of cancer survivors: those who had undergone rapid orthodontic treatment (up to 12 months) and standard orthodontic treatment (orthodontic treatment time longer than 16 months).

There have been described several skeletal and dental complications, as well as complications related to oral mucous membrane in patients who had undergone oncological treatment in their childhood [23,24,25,26,27]. The above-mentioned complications included hypodontia, microdontia, root malformation, enamel defects, growth and developmental changes, as well as oral mucositis [23,24,25,26,27]. Sonis et al. [26] found that 94% of cancer survivors diagnosed with cancer below the age of 5 years old presented disturbed dental development. Moreover, children who had undergone oncological treatment below the age of 5 years old and those who had received radiotherapy, presented even more severe dental abnormalities. Kılınç et al. [25] presented similar observations. The authors classified the patients who had received oncological treatment below the age of 7 years old as a high-risk group for dental abnormalities. Furthermore, they also noticed that the incidence rates of microdontia and hypodontia increased when the patients had been treated oncologically below the age of 5 years old. Although, in our study, the mean age at oncological diagnosis was below 5 years old for each type of cancer in both groups, we did not observe any other dental abnormalities apart from root resorption. There were only four patients diagnosed with root resorption in the group with standard duration of orthodontic treatment. None of the patients from the rapid group presented any signs of root resorption. All our patients had been treated with chemotherapy, and four patients had received additional radiotherapy (two patients in each group). None of the chemoradiotherapy patients had received radiotherapy in the area of the head and neck, which could have explained why those patients had not developed the dental abnormalities described by other authors.

Disturbances in the growth of the craniofacial skeleton as consequences of oncologic treatment were also discussed in the literature [8,23,26]. Changes in skeletal growth were associated most often with radiotherapy. Sonis et al. [26] noticed that patients who had received chemotherapy with additional 2400 cGy radiotherapy in the area of head below the age of 5 years old, presented significantly deficient mandibular growth. Roman et al. [27] found that chemotherapy as a sole method of oncologic treatment in children interfered with growth, both during and after treatment. The authors noticed that chemotherapy provokes growth hormone deficiency. Although, the exact impact of chemotherapy alone on mandibular growth has not been assessed yet, it may be speculated that if the chemotherapy alone provokes growth hormone deficiency, it could also lead to mandibular growth deficiency. The majority of cancer survivors included into our study had skeletal class II with deficient mandibular growth: 55.6% in rapid group and 55.0% in standard group. However, in our opinion, skeletal class II in those patients cannot be considered as a direct consequence of chemotherapy alone, because of two facts. Firstly, skeletal class II is a very common type of malocclusion in Poland, and secondly, there were also cancer survivors included in the study who were diagnosed with moderate skeletal class III with excessive mandibular growth and skeletal class I with optimal anteroposterior position of the maxilla and mandible.

Oral mucositis has been described as the acute reaction in the area of the oral cavity mucous membrane, which occurs during chemoradiotherapy. Its incidence seems to be underreported by oncologists [28,29]. There are five stages of mucositis progression: initiation, the primary damage response, amplification, ulceration, and healing. The initiation phase is characterized not only by direct DNA damage but also by the generation of reactive oxygen species [28]. Pain associated with oral mucositis was found to impair the function of the oral cavity: phonation, deglutition, and dysgeusia [29]. Finally, it was found that oral mucositis significantly diminished OHRQoL in patients diagnosed with cancer [29]. We have found that seven patients (17.5%) in the standard group and six patients (16.7%) in the rapid suffered from oral mucositis. There were no significant differences between the examined groups regarding the incidence of oral mucositis.

Apart from the abovementioned complications, one of our patients needed hyperbaric oxygen support after the extraction of the lower left third molar. According to the literature [30], complications post extractions in cancer patients ranged from 3% to 40%, and they occurred most commonly after third molar extraction, which stays in agreement with our observation. Although, the weighted prevalence of dental infections during cancer therapy is rather low (5.4%), use of fluoride and use of chlorhexidine are recommended, especially after radiotherapy in the area of head and neck [30].

Quality of life among children and adolescents with cancer was found to be associated with several variables, including general fatigue, sleep fatigue, cognitive fatigue, self-concern distress, physical and psychological distress, school life distress, relationship distress, family life distress, age at diagnosis, time since diagnosis, and family structure. Four major predictors of quality of life encompass general fatigue, relationship distress, nuclear family, and time since diagnosis [31].

According to the literature, malocclusion has an impact on OHRQoL. It has been proven that with the increased severity of the malocclusion and with more severely compromised esthetics, patients experience lower OHRQoL [15,32]. Moreover, among different types of malocclusion, the most significant impacts on OHRQoL were caused by impacted canine, increased overjet, and displacement of teeth [32]. Not only the type of malocclusion but also the orthodontic treatment by itself has an impact on OHRQoL. Demirovic et al. [33] found that patients who had completed the orthodontic treatment had better OHRQoL than those who had never undergone orthodontic treatment. Moreover, adults who required both orthodontic treatment and orthognathic surgery achieved the biggest improvement in OHRQoL, while the least improvement in OHRQoL was observed in patients with developmental abnormalities, including cleft lip, cleft palate, or a combination of both [34]. Nascimento et al. [35] concluded that orthodontic treatment provided psychological benefits, because it led to a significant increase in self-esteem and improvement in the quality of life. Our results also supported these observations. Patients from both groups presented significant improvement of the values of oral-health-related quality of life at the end of orthodontic treatment comparing to the values achieved before the onset of orthodontic treatment. Although, the participants treated orthodontically with the rapid protocol achieved slightly better results regarding the OHIP-14 mean total score at the end of orthodontic treatment, there were no significant differences between the examined groups. These results indicate that the duration of orthodontic treatment does not have a significant impact on the oral-health-related quality of life in cancer survivors.

Besides the above-mentioned observations, we have also found that oral-health-related quality of life significantly decreased after two weeks and three months after the onset of orthodontic treatment. The OHIP-14 mean total score values were significantly higher two weeks and three months after the onset of orthodontic treatment comparing to the values obtained before the orthodontic treatment. This was mostly the result of increased physical pain within the first three months of orthodontic treatment. Moreover, the OHIP-14 mean total score values achieved three months after the onset of the orthodontic treatment were significantly lower comparing to the values obtained two weeks after the onset of the orthodontic treatment. These results indicate that although oral-health-related quality of life significantly decreased after bonding the fixed appliances, it also improved after a few months of orthodontic treatment, which may be explained that the patients got used to the inconvenience related to orthodontic therapy. Similar results presenting significant decrease of oral-health-related quality of life within the first three months of orthodontic treatment were also obtained by Johal et al. [17]. However, the authors did not find any significant differences in oral-health-related quality of life between before and after the end of orthodontic treatment.

There are several limitations to our study. First of all, there was a limited number of participants included into the study. The rapid group consisted of 36 cancer survivors, whereas the standard group consisted of 40 cancer survivors. Secondly, the median age was under 20 years old. Therefore, the obtained results should not be generalized for the elderly population of cancer survivors. Thirdly, cancer survivors were not allocated into the groups at random but on the basis of the result of specialist oncological consultation. Patients, who were allocated into the rapid orthodontic group, were at risk of possible hospitalization, magnetic resonance imaging examination, or any other types of oncological examinations that could have influenced orthodontic treatment within the following one year. It may be speculated that although cancer survivors from the rapid group finished their orthodontic treatment earlier than the standard group, they could have been full of fears regarding the above-mentioned hospitalization or additional examinations, which could have had a direct impact on their general quality of life. Fourthly, although we did our best to perfectly match the groups regarding the type of malocclusion, there were some insignificant differences between the groups. Moreover, it should be clearly stated that even if different types of malocclusion seem to be very similar, they are never the same ones. One of the most important aspects, that influences the course of orthodontic treatment is the biological response to the orthodontic mechanics.

## 5. Conclusions

The results of the orthodontic treatment obtained in both groups were similar, although one of the groups had received shorter orthodontic treatment. Duration of orthodontic treatment did not influence the oral-health-related quality of life in cancer survivor patients. Orthodontic treatment had a positive impact on oral-health-related quality of life in both groups of cancer survivors. However, within first three months after the onset of orthodontic treatment, significant worsening of oral-health-related quality of life was observed. Rapid orthodontic treatment can be completed successfully in cancer survivors without any negative impact on oral-health-related quality of life or on the general outcome of the treatment.

## Figures and Tables

**Table 1:** General characteristics of cancer survivors and control group of patients.

Factor	Rapid Group	Standard Group	*p*-Value ^a^
Number of patients (*n*) (female/male ratio)	36 (26/10)	40 (22/18)	0.746
Median age (range) (years)	19.4 (13–28)	19.2 (14–28)	0.811
Orthodontic assessment (number of patients)	Skeletal class I: 10 Skeletal class II: 20 Skeletal class III: 6	Skeletal class I: 14 Skeletal class II: 22 Skeletal class III: 4	0.640

^a^ U Mann–Whitney test.

**Table 2:** General characteristics of both rapid and standard groups with reference to the diagnosed type of cancer, including mean age at diagnosis, follow-up time, and treatment modality.

Diagnosis	Number of Cases	Mean Age at Diagnosis [Years]	Follow-Up Time [Years]	Treatment Modality	
				Chemotherapy	Radiotherapy
Rapid Group	36				
Leukemia	22	3.9 ± 1.5	8.8 ± 4.2	22	0
Neuroblastoma	3	0.8 ± 0.5	4.1 ± 1.2	3	0
Soft tissue sarcoma	2	2.5 ± 1.1	6.4 ± 2.4	2	1
Non-Hodgkin’s lymphoma	6	4.9 ± 2.4	7.3 ± 3.3	6	1
Wilms’ tumor	3	3.2 ± 2.2	6.8 ± 3.1	3	0
Standard Group	40				
Leukemia	25	3.2 ± 1.2	9.8 ± 3.7	25	0
Neuroblastoma	4	0.8 ± 0.5	5.4 ± 1.2	4	0
Soft tissue sarcoma	3	2.2 ± 0.8	7.5 ± 2.2	3	1
Non-Hodgkin’s lymphoma	2	4.6 ± 2.4	7.6 ± 3.9	2	1
Wilms’ tumor	6	3.6 ± 2.3	6.6 ± 2.9	6	0

**Table 3:** The 14-item Oral Health Impact Profile (OHIP-14) Questionnaire [18,20].

The List of Questions in the OHIP-14 Questionnaire
Functional Limitation
1. Have you had trouble pronouncing any words because of problems with your teeth or mouth? 2. Have you felt that your sense of taste has worsened because of problems with your teeth or mouth?
Physical Pain
3. Have you had painful aching in your mouth? 4. Have you found it uncomfortable to eat any foods because of problems with your teeth or mouth?
Psychological Discomfort
5. Have you been self-conscious because of your teeth or mouth? 6. Have you felt tense because of problems with your teeth or mouth?
Physical Disability
7. Has been your diet been unsatisfactory because of problems with your teeth of mouth? 8. Have you had to interrupt meals because of problems with your teeth or mouth?
Psychological Disability
9. Have you found it difficult to relax because of problems with your teeth or mouth? 10. Have you been a bit embarrassed because of problems with your teeth or mouth?
Social Disability
11. Have you been a bit irritable with other people because of problems with your teeth or mouth? 12. Have you had difficulty doing your usual jobs because of problems with your teeth or mouth?
Handicap
13. Have you felt that life in general was less satisfying because of problems with your teeth or mouth? 14. Have you been totally unable to function because of problems with your teeth or mouth?

**Table 4:** The OHIP-14 Mean Total Score in the cancer survivors treated orthodontically according to the rapid and standard protocols before, during, and after orthodontic treatment.

Time of Orthodontic Treatment (TX)	Rapid Group (Mean ± SD) (Range)	Standard Group (Mean ± SD) (Range)	*p*-Value ^a^
Before TX	4.1 ± 4.2 ^1,2,3^ (0–14)	3.8 ± 3.0 ^4,5,6^ (0–9)	0.311
2 weeks after the onset of TX	9.8 ± 8.2 ^1,7,8^ (0–32)	10.2 ± 6.8 ^4,10,11^ (0–20)	0.189
3 months after the onset of TX	7.8 ± 7.5 ^2,7,9^ (0-28)	8.5 ± 4.3 ^5,10,12^ (0–15)	0.324
After TX	1.1 ± 2.8 ^3,8,9^ (0–14)	1.3 ± 2.3 ^6,11,12^ (0–12)	0.108

^1–12^ Statistically significant differences with *p* < 0.001 (ANOVA), ^a^ Mann–Whitney U test, SD—standard deviation.

**Table 5:** The mean OHIP-14 score for individual domains observed before, during, and after orthodontic treatment for the two groups of cancer survivors: with rapid and standard duration of orthodontic treatment.

OHIP-14 Domains	Functional Limitation	Physical Pain	Psychological Discomfort	Physical Disability	Psychological Disability	Social Disability	Handicap
Before	0.3 ± 0.6	0.1 ± 0.4	0.4 ± 0.7	0.7 ± 1.1	0.7 ± 1.2	0.4 ± 0.5	0.8 ± 1.3
(rapid vs. standard)	0.3 ± 0.5	0.1 ± 0.2	0.2 ± 0.5	0.6 ± 0.7	0.8 ± 1.1	0.3 ± 0.6	0.5 ± 1.2
2 weeks	0.8 ± 0.8	2.1 ± 2.5	1.3 ± 1.9	1.6 ± 1.4	1.2 ± 1.4	0.4 ± 0.7	0.7 ± 1.2
(rapid vs. standard)	0.9 ± 1.3	1.8 ± 2.1	1.1 ± 0.5	1.4 ± 1.1	1.0 ± 1.2	0.4 ± 0.5	0.6 ± 1.4
3 months	0.3 ± 0.8	1.3 ± 2.3	1.0 ± 1.5	1.4 ± 1.7	1.0 ± 1.3	0.4 ± 0.5	0.4 ± 1.2
(rapid vs. standard)	0.5 ± 0.8	1.1 ± 0.8	0.9 ± 1.3	1.2 ± 1.1	1.2 ± 0.9	0.2 ± 0.5	0.5 ± 1.8
After	0.1 ± 0.4	0.2 ± 0.5	0.2 ± 0.6	0.3 ± 0.6	0.1 ± 0.5	0.1 ± 0.4	0.1 ± 0.2
(rapid vs. standard)	0.1 ± 1.1	0.1 ± 0.3	0.1 ± 0.5	0.2 ± 0.4	0.1 ± 0.4	0.2 ± 0.3	0.1 ± 0.6

**Table 6:** The number of patients with impaired domains in cancer survivors with rapid and standard duration of orthodontic treatment.

OHIP-14 Domains	Functional Limitation	Physical Pain	Psychological Discomfort	Physical Disability	Psychological Disability	Social Disability	Handicap
Before	2	0	6	8	8	7	8
(rapid vs. standard)	3	0	4	6	6	6	7
2 weeks	4	11	18	12	13	5	6
(rapid vs. standard)	4	12	16	11	11	4	5
3 months	7	10	7	9	9	5	5
(rapid vs. standard)	7	8	8	8	7	3	3
After	1	0	2	2	1	1	1
(rapid vs. standard)	0	1	1	1	1	1	0

**Table 7:** OHIP-14 Simple Count—the number of patients with oral health impacts reported as occasionally or more frequently.

Time of Orthodontic Treatment (TX)	Rapid Group	Standard Group	*p*-Value ^a^
Before	3 (8.3%)	3 (7.5%)	0.965
2 weeks	17 (47.2%)	18 (45.0%)	0.312
3 months	12 (33.3%)	12 (30.0%)	0.845
After the treatment	2 (5.6%)	1 (2.5%)	0.514

^a^ Chi-square test.
